# Neurotensin Agonist Attenuates Nicotine Potentiation to Cocaine Sensitization

**DOI:** 10.3390/bs4010042

**Published:** 2014-01-22

**Authors:** Paul Fredrickson, Mona Boules, Bethany Stennett, Elliott Richelson

**Affiliations:** Neuropsychopharmacology Laboratory, Mayo Foundation for Medical Education and Research Mayo Clinic Florida, 4500 San Pablo road, Jacksonville, FL 32224, USA; E-Mails: boules.mona@mayo.edu (M.B.); richel@mayo.edu (E.R.); bastenn@gmail.com (B.S.)

**Keywords:** neurotensin, nicotine, cocaine, gateway drug, rat

## Abstract

Tobacco usage typically precedes illicit drug use in adolescent and young adult populations. Several animal studies suggest nicotine increases the risk for subsequent cocaine abuse, and may be a negative prognostic factor for treatment of cocaine addiction; *i.e.*, a “gateway drug”. Neurotensin (NT) is a 13-amino acid neuropeptide that modulates dopamine, acetylcholine, glutamate, and GABA neurotransmission in brain reward pathways. NT69L, a NT(8-13) analog, blocks behavioral sensitization (an animal model for psychostimulant addiction) to nicotine, and nicotine self-administration in rats. The present study tested the effect of NT69L on the potentiating effects of nicotine on cocaine-induced locomotor sensitization. Male Wistar rats were injected daily for seven days with nicotine or saline (control) followed by four daily injections of cocaine. NT69L was administered 30 min prior to the last cocaine injection. Behavior was recorded with the use of activity chambers. Subchronic administration of nicotine enhanced cocaine-induced behavioral sensitization in Wistar rats, consistent with an hypothesized gateway effect. These behavioral effects of cocaine were attenuated by pretreatment with NT69L. The effect of the neurotensin agonist on cocaine sensitization in the nicotine treated group indicated a possible therapeutic effect for cocaine addiction, even in the presence of enhanced behavioral sensitization induced by nicotine.

## 1. Introduction

Cigarette smoking has been conceptualized as a gateway to use and abuse of illicit drugs such as cocaine [1]. Whether this is causal or represents availability and cultural attitudes is as yet unproven [2]. However, animal models provide growing evidence for a “priming effect” of nicotine on reward circuitry. This priming effect may increase the risk for cocaine abuse and dependence [3,4]. 

Animal models of psychostimulant abuse include behavioral sensitization, conditioned place preference, drug discrimination, and self-administration [5]. Nicotine pretreatment enhances cocaine self-administration in adolescent [6,7,8] and in adult [9] rats. Nicotine also increases cocaine’s discriminative stimulus and reinforcing effects in rhesus monkeys [10]. In another recent study [4], locomotor sensitization was increased by 98%, conditioned place preference by 78%, and cocaine-induced reduction in long-term potentiation was increased by 24% when nicotine was given prior to, and then concurrent with cocaine. Reversing the order of drug administration had no effect. Therefore, nicotine primed the response to cocaine, but cocaine did not have a similar effect on nicotine-induced behaviors or synaptic plasticity.

These animal studies suggest, although do not prove, that tobacco users are at increased risk for cocaine dependence, and that continued use of nicotine may be a negative prognostic factor for abstinence from cocaine. Furthermore, these studies point toward a need for therapies that target both nicotine and cocaine dependence. For the past decade, our laboratory has studied the effects of neurotensin (NT) analogs on neuropsychiatric diseases including psychostimulant abuse. One of our analogs, NT69L, an analog of NT(8-13), blocks the initiation and expression of nicotine-induced locomotor sensitization [11], and attenuates nicotine self-administration in rats [12]. 

NT is a neuropeptide that is closely associated with, and modulates DA [13], ACh, glutamate, and GABA neurotransmission involved in addiction and reward pathways. Local administration of NT in the prefrontal cortex (PFC) increases extracellular levels of ACh and GABA [14]. In support of these data are results with the NT agonist NT69L [15] which was developed in our laboratory. NT has also been reported to enhance GABAergic activity in rat hippocampus [16] and to reduce glutamatergic neurotransmission in dorsolateral striatum [17]. However, NT must be administered centrally to have an effect because it is easily degraded by peptidases. Our laboratory has developed a number of NT agonists that can be administered systemically and maintain the central effects of NT. The most studied of these agonists is NT69L which binds with equal affinity to the two well characterized NT receptors (NTS1 and NTS2).

Our work with the NT agonist NT69L shows that it blocks nicotine-induced sensitization [11,18] and nicotine self-administration [12]. While the role of NT is strongly implicated in nicotine addiction, its role in cocaine addiction is controversial. Administration of cocaine increases NT immunoreactivity in the dorsal striatum, substantia nigra and globus pallidus [19], increases NT mRNA in the rostral striatum [20] and increases NT gene expression in the ventrolateral striatum [21]. The use of NT gene knockout mice showed that NT is not involved in cocaine mediated behavior except under certain conditions where the absence of endogenous NT causes a slight prolongation of the effects of cocaine [22]. The use of either NT agonists or antagonists to treat cocaine locomotor activity and locomotor sensitization gave mixed results. Administration of the NT antagonist SR48692 did not alter cocaine-induced locomotor activity [23,24] except when administered chronically and in high doses [24]. Others reported partial reversal of the expression of locomotor sensitization with SR48692 [25] or a delay in the development of cocaine sensitization when the SR compound is given prior to but not in conjunction with cocaine [26]. There is also mixed data with the use of NT agonists. Systemic administration of the nonselective NT agonist NT69L [27] or central administration of NT [24] attenuate cocaine-induced locomotor activity. However, central administration of NT(8-13) did not alter cocaine-induced locomotor activity or cocaine sensitization [28]. Biochemically, NT [24] and NT69L [29] interact with dopamine, the neurotransmitter that promotes the motivational process for both nicotine and cocaine.

The primary goal of the present study was to examine the effect of the neurotensin receptor agonist, NT69L, on nicotine-induced potentiation of cocaine locomotor sensitization.

## 2. Methods

### 2.1. Animals

Male Wistar rats (n = 4–16 per group) weighing 200–220 g at the start of the experiments were used. Animals were housed in temperature controlled rooms with free access to food and water. All animal procedures were approved by Mayo Clinic Institutional Animal Care and Use Committee. 

### 2.2. Treatments

**Figure 1:**
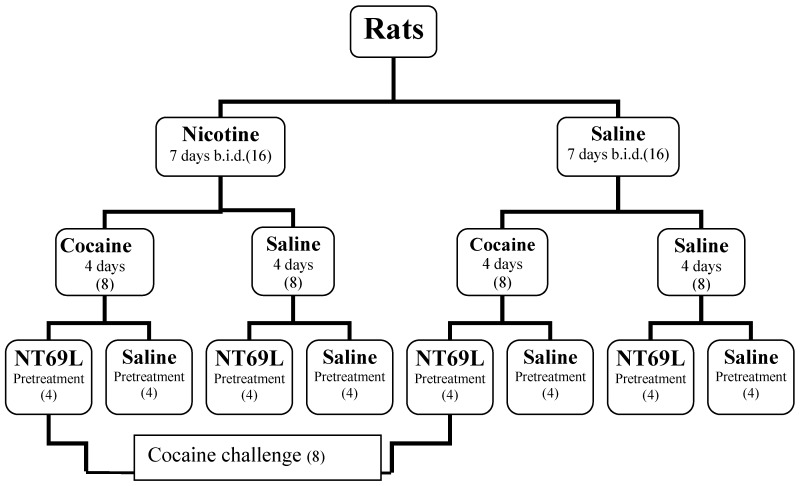
Experimental design.

The rats were divided into 2 groups (n = 16 per group) (Figure 1). One group was injected with nicotine (0.35 mg/kg, s.c.) twice daily, (8:30 AM and 4:30 PM) for 7 days and the other group was injected at the same time with saline. Each group was then subdivided into 2 groups. One group was injected with cocaine (20 mg/kg i.p.) and the other with saline daily for four days. The effect of NT69L (1 mg/kg i.p.) was tested in half the animals in each group. To test for the effect of NT69L on cocaine challenge, the cocaine-treated animals in both the nicotine and the saline groups were given one dose of cocaine (20 mg/kg i.p.) 48 h after the last cocaine injection. Figure 1 shows a summary of the treatments.

### 2.3. Behavioral Testing

Locomotor activity was determined in a Plexiglas Opto-Varimex activity chamber (Columbus, OH, USA) equipped with infrared photocell emitters and detectors. The rats were acclimated in the room for 1 h and then placed in the activity chambers. Baseline activity was recorded for 1 h after which animals were injected with saline, nicotine, or cocaine. Activity was recorded as distance travelled in cm every 10 min in a 2 h period. To test the effects of NT69L, animals were injected with NT69L (1 mg/kg i.p.) 30 min prior to the injection of cocaine.

(n) = number of animals per group.

### 2.4. Drugs

NT69L was synthesized by Mayo Clinic Chemistry Core facility as described previously [30]. NT69L was administered at a dose of 1 mg/kg i.p. The dose of NT69L was based on results from our previous studies [12,27,29]

Nicotine hydrogen tartrate salt and cocaine were purchased from Sigma Chemical Co. (St Louis, MO, USA) and were dissolved in sterile physiological saline (0.9% sodium chloride). Nicotine was administered at a dose of 0.35 mg/kg s.c., and cocaine was administered at a dose of 20 mg/kg i.p., based on results from on our previous studies for nicotine [11,18] and for cocaine [27].

### 2.5. Statistical Analysis

One-way analysis of variance (ANOVA) was used to analyze the data. Individual group comparisons were carried out using the Holm-Sidak test with the use of SigmaStat 2.0 software (SPSS, Inc., Chicago, IL, USA), with *P* < 0.05 being considered significant.

## 3. Results

There was no significant difference in locomotor activity between animals treated with saline once (acute) or daily for seven days (*P* = 0.428), thus the two groups were averaged and depicted as “saline” group.

### 3.1. Effect of Nicotine on Locomotor Activity

**Figure 2:**
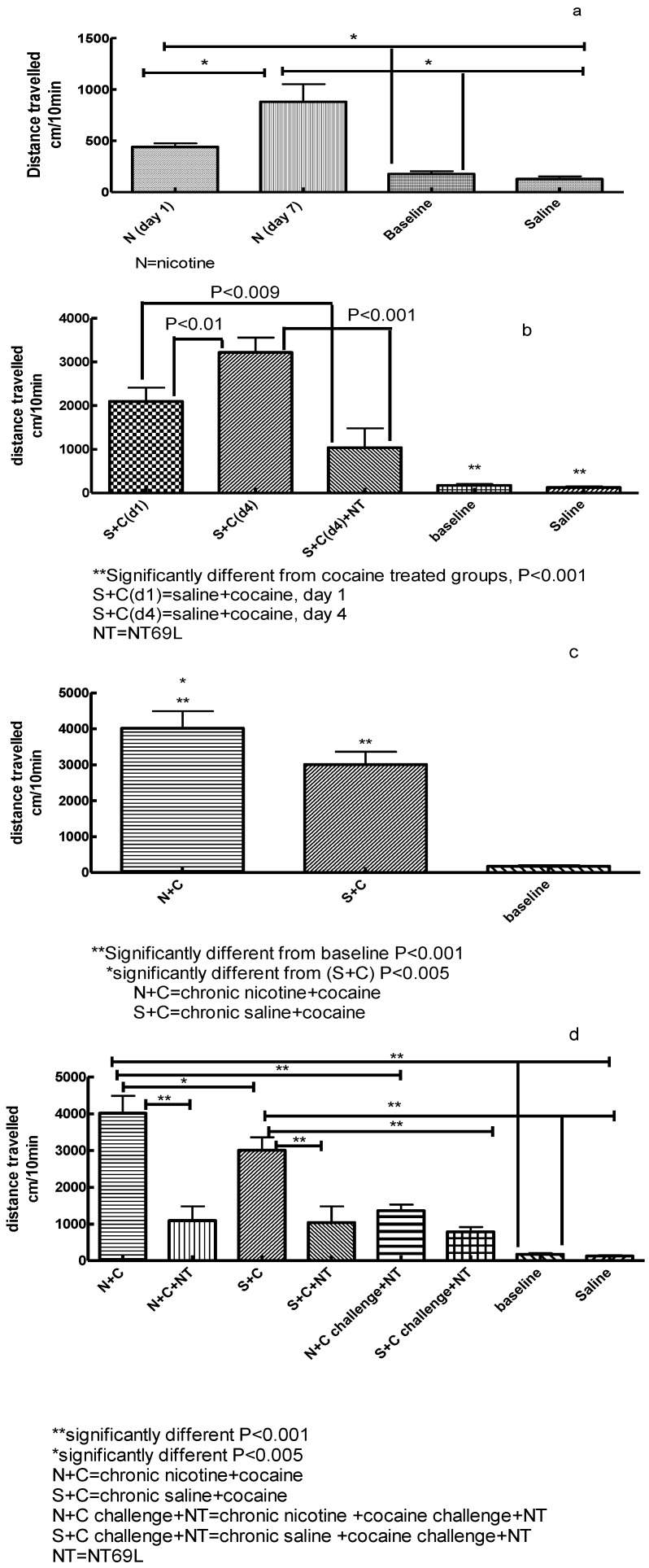
Effects of NT69L on locomotor activity in rats exposed to nicotine and cocaine. Male Wistar rats (n = 4-16 per group) were injected twice daily for seven days with nicotine (0.35 mg/kg s.c.) or saline control followed by four daily injections of cocaine (20 mg/kg i.p.). Cocaine challenge (20 mg/kg i.p.) was administered 48 h after the last cocaine injection. NT69L (1 mg/kg i.p.) was administered 30 min prior to cocaine injection on test days. Behavior was recorded with the use of activity chambers. (**a**) Effect of chronic nicotine injection on locomotor activity; (**b**) Effect of NT69L on cocaine-induced locomotor sensitization; (**c**) Effect of chronic nicotine pretreatment on subsequent cocaine sensitization; (**d**) Effect of NT69L on expression of nicotine-enhanced cocaine sensitization and cocaine challenge.

Acute and 7 daily treatments twice per day with nicotine (0.35 mg/kg s.c.) significantly (F_4,46_ = 11.339, *P* < 0.001) increased locomotor activity. Injection of nicotine significantly increased locomotor activity as compared to baseline and saline control, *P* < 0.05. Repeated injection of nicotine further increased locomotor activity as compared to acute injection (*P* < 0.03), results that are indicative of sensitization (Figure 2a).

### 3.2. Effect of NT69L on Cocaine-Induced Sensitization

One way ANOVA show that cocaine treatment (20 mg/kg i.p.) significantly increased locomotor activity (F_5,47_ = 43.462, *P* < 0.001). Acute injection of cocaine significantly increased locomotor activity as compared to baseline and saline controls (*P* < 0.001). Daily injection of cocaine for four days further increased locomotor activity, results that are indicative of sensitization (*P* < 0.01). Pretreatment of cocaine-sensitized rats with NT69L (1 mg/kg i.p.) attenuated cocaine-induced locomotor activity (*P* < 0.001) (Figure 2b).

### 3.3. Effect of Chronic Nicotine Treatment on Subsequent Cocaine Sensitization

One-way ANOVA (F_2,24_ = 52.933, *P* < 0.001) show that pretreatment with nicotine (0.35 mg/kg s.c.) twice daily for seven days significantly increased cocaine-induced locomotor sensitization as compared to saline pretreated animals (*P* < 0.032). As expected, injection of cocaine (20 mg/kg i.p.) in the nicotine and saline pretreated animals, significantly increased locomotor activity as compared to baseline activity (*P* < 0.001), Figure 2c.

### 3.4. Effect of NT69L on Expression of Nicotine-Enhanced Cocaine-Induced Sensitization and Cocaine Challenge

One-way ANOVA show significant effect of treatment (F_7,55_ = 40.332, *P* < 0.001). Further pairwise comparisons show that daily administration of nicotine significantly increased cocaine-induced locomotor sensitization (*P* < 0.005) as compared with daily saline. Pretreatment with NT69L (1 mg/kg i.p.) significantly attenuated cocaine-induced locomotor activity in both the nicotine- (*P* < 0.001) and the saline- (*P* < 0.001) pretreated animals. Additionally, NT69L markedly reduced locomotor activity after cocaine challenge in both the nicotine- (*P* < 0.001) and the saline- (*P* < 0.001) pretreated animals. All nicotine and cocaine treated animals showed increased locomotor activity as compared to baseline activity (prior to treatment) and saline treated animals (*P* < 0.001). There was no significant difference between saline treated animals and baseline activity of all tested animals (*P* = 0.973), Figure 2d.

## 4. Discussion

Locomotor sensitization is a robust and readily assayed effect of psychostimulants. It measures the animal’s gradually increasing behavioral and motivational response to a fixed drug dose, assayed as an increase in locomotor activity [4] (for review, Robinson and Berridge, [31]). Locomotor sensitization has been shown in rodents to be associated with augmented drug reward and increased vulnerability to relapse [31].

Locomotor sensitization is separated into two components: induction and expression. Induction of sensitization is the progressive increase in locomotor activity during drug treatment, while the expression of sensitization is demonstrated following challenge with cocaine after a drug-free period [32]. Our results show that the rats developed locomotor sensitization after repeated injections of nicotine, as we previously showed [11]. Additionally, and similar to previous studies [7,8,33,34], both induction and expression of locomotor sensitization were observed in the present study after four daily injections of cocaine and cocaine challenge. Our group has reported that NT69L blocks the initiation and expression of nicotine sensitization [11], as well as nicotine self-administration [12]. 

In the present study NT69L attenuated the expression of cocaine sensitization. Sub-chronic injection of nicotine enhanced cocaine-induced sensitization, results that are consistent with the “gateway” effect of nicotine on cocaine abuse [4]. Prior studies showed cross-sensitization of nicotine and cocaine-induced locomotion [35,36]. In a study of adolescent rats designed to model smoking behaviors of human adolescents, injections of low dose nicotine (0.06 mg/kg) did not produce behavioral sensitization. However, low dose nicotine did enable locomotor sensitization to cocaine that was not seen in control animals [8]. The difference in the results between our study and that of the McQuown group might be due to: (1) difference in the rat species used; (2) age difference in animals (adolescent *versus* adults); (3) dose and route of administration of nicotine; and (4) dose and route of administration of cocaine. The effect of NT69L on cocaine sensitization in the nicotine treated group indicates its possible therapeutic effect even with the enhanced locomotor sensitization induced by nicotine.

Cocaine dependence results from drug-induced neural adaptations in mesocorticolimbic dopamine pathways and associated glutamatergic circuitry [37]. Induction of sensitization requires activation of dopaminergic and glutamatergic neurotransmission in the ventral tegmental area [38,39] and glutamatergic input from the prefrontal cortex [40,41]. Expression of sensitization is primarily attributed to neurochemical changes in the nucleus accumbens [42] leading to cocaine-induced increase in dopamine and glutamate release in the nucleus accumbens [34,43,44].

Our previous studies [29] show that pretreatment with NT69L attenuates the acute nicotine evoked increases in dopamine in the nucleus accumbens shell and reverses the increase in dopamine levels in the nucleus accumbens core. NT69L also modulates tyrosine hydroxylase, dopamine D1 and D2 receptor mRNA levels in the striatum, prefrontal cortex, and ventral tegmental area in rats that self-infused nicotine [12]. In addition to modulating dopaminergic neurotransmission, NT69L modulates the glutamatergic neurotransmission that is involved in the addiction of cocaine and other drugs such as alcohol ([45]; Boules *et al*., unpublished data).

NT69L’s attenuation of some of the biochemical effects of acute and chronic nicotine is consistent with this peptide's attenuation of nicotine-induced behavioral effects. Findings by Levine *et al*. [4] suggest that nicotine replacement therapy given to cocaine addicts for smoking cessation may undermine efforts to treat cocaine addiction. Novel therapies, such as neurotensin analogs, therefore, warrant additional study.

## 5. Conclusions

Subchronic administration of nicotine enhanced cocaine-induced behavioral sensitization in Wistar rats, consistent with an hypothesized gateway effect. These behavioral effects of cocaine were attenuated by pretreatment with NT69L. The data presented here are further evidence for a role for NT69L or other neurotensin receptor agonists to treat nicotine addiction, and provide preliminary support that these compounds may be useful for management of cocaine addiction.
